# Antidepressant Response in Major Depressive Disorder: A Meta-Regression Comparison of Randomized Controlled Trials and Observational Studies

**DOI:** 10.1371/journal.pone.0020811

**Published:** 2011-06-08

**Authors:** Florian Naudet, Anne Solène Maria, Bruno Falissard

**Affiliations:** 1 Institut National de la Santé Et de la Recherche Médicale U669, Paris, France; 2 Université de Rennes 1, Unité de Recherche Universitaire EM-425 Behavior and Basal Ganglia, Rennes, France; 3 Centre Hospitalier Guillaume Régnier, Service Hospitalo-Universitaire de Psychiatrie, Rennes, France; 4 Ecole de Psychologues Praticiens, Paris, France; 5 Université Paris-Sud and Université Paris Descartes, Unité Mixte de Recherche S0669, Paris, France; 6 Assistance Publique Hôpitaux de Paris, Hôpital Paul Brousse, Département de santé publique, Villejuif, France; Chiba University Center for Forensic Mental Health, Japan

## Abstract

**Background:**

To compare response to antidepressants between randomized controlled trials (RCTs) and observational trials.

**Methods and Findings:**

Published and unpublished studies (from 1989 to 2009) were searched for by 2 reviewers on Medline, the Cochrane library, Embase, clinicaltrials.gov, Current Controlled Trial, bibliographies and by mailing key organisations and researchers. RCTs and observational studies on fluoxetine or venlafaxine in first-line treatment for major depressive disorder reported in English, French or Spanish language were included in the main analysis. Studies including patients from a wider spectrum of depressive disorders (anxious depression, minor depressive episode, dysthymia) were added in a second analysis. The main outcome was the pre-/post-treatment difference on depression scales standardised to 100 (17-item or 21-item Hamilton Rating Scale for Depression or Montgomery and Åsberg Rating Scale) in each study arm. A meta-regression was conducted to adjust the comparison between observational studies and RCTs on treatment type, study characteristics and average patient characteristics. 12 observational studies and 109 RCTs involving 6757 and 11035 patients in 12 and 149 arms were included in the main analysis. Meta-regression showed that the standardised treatment response in RCTs is greater by a magnitude of 4.59 (2.61 to 6.56). Study characteristics were related to standardised treatment response, positively (study duration, number of follow-up assessments, outpatients versus inpatients, per protocol analysis versus intention to treat analysis) or negatively (blinded design, placebo design). At patient level, response increased with baseline severity and decreased with age. Results of the second analysis were consistent with this.

**Conclusions:**

Response to antidepressants is greater in RCTs than in observational studies. Observational studies should be considered as a necessary complement to RCTs.

## Introduction

Antidepressant drugs have become the cornerstone of the treatment of major depressive disorder (MDD). Recently, three meta-analyses questioned this picture, emphasising the number of non-published negative studies [1] and the importance of the placebo response in mild to moderate depressive disorders [2], [3]. Placebo response has increased significantly in recent years [4], it has been related to intensive follow-up by trained teams [5], linked to the probability of receiving a placebo [6], and found to depend on the characteristics of the population included [7]. To cope with this phenomenon, some randomized controlled trials (RCTs) exclude placebo responders on the basis of a one-week placebo run-in period [8], and use strict inclusion criteria [9].

Thus, experimental conditions that enhance internal validity to prove antidepressant efficacy can modify the effect demonstrated. They do not correspond to antidepressant use in real life [10], and decrease the external validity of such studies.

The determination of effectiveness is part of the post-listing assessment process, via observational studies. In the cardiovascular field, some authors have worked on the link between randomized controlled trials and observational studies [11]; in psychiatry this has been applied to psychotherapy [12], but, to our knowledge, nobody has explored this issue for antidepressants.

To quantify the links between antidepressant efficacy and effectiveness we reviewed RCTs and observational trials in MDD first line treatment using fluoxetine, the first selective serotonin reuptake inhibitor available on the market which has become a reference drug and venlafaxine a serotonin-norepinephrine reuptake inhibitor which was the first antidepressant in terms of sales in 2008 [13]. The main objective was to compare observed response to antidepressants in RCTs with response in observational trials. Over a wide range of antidepressant trials, the secondary objective was to synthesise and quantify the impact of all methodological choices on the measurement of antidepressant response: blind design, placebo design, year of publication, number of follow up assessments, type of analysis, exclusion of placebo responders and patients' characteristics as baseline severity.

## Methods

The methods of this meta-analysis on aggregated data and the inclusion criteria were pre-specified and documented in a written protocol.

### Eligibility criteria

#### Types of participants

In the main analysis, we reviewed studies involving adults with a diagnosis of MDD (DSM IV, DSM IV-R, DSM III, DSM III-R, ICD 10, Feighner criteria, Research Diagnostic Criteria). Studies involving patients with other psychiatric or medical comorbidities were considered, except if these comorbidities were an explicit inclusion criterion for the study. Studies involving more than 20% bipolar disorder were excluded, as were studies exclusively involving elderly patients or patients with seasonal affective disorder, post partum depression, postmenopausal depression, atypical depression.

As in “real-life” a wide range of depressive disorders is treated with antidepressants, a second analysis included studies involving patients with a diagnosis of anxious depression (criteria for both an anxious disorder and MDD) and/or minor depressive episode and/or dysthymia.

#### Types of intervention

We focused our attention on fluoxetine and venlafaxine in oral mono-therapy for MDD first-line treatment. By choosing these two antidepressants, which are widely used, we were sure to have a large number of RCTs and observational studies.

#### Types of outcome

The primary outcome measure was the difference between baseline and last assessment on the 17-item or 21-item Hamilton Rating Scale for Depression (HRSD) or the Montgomery and Åsberg Rating Scale (MADRS).

Studies not providing the desired information on these scales were included in the qualitative review.

#### Types of study

In this review the studies considered were those designed to measure antidepressant efficacy or effectiveness, conducted between January 1989 and July 2009: on the one hand RCTs (antidepressant versus placebo or active treatment) and on the another hand observational cohorts (longitudinal non-randomized and non-blinded studies). Studies designed to provide evidence on other issues such as physiological hypotheses were not retained. Only study reports in English, French and Spanish language were considered.

### Search strategy

Eligible studies were identified from Pubmed/Medline, the Cochrane library, and Embase, including conference abstracts. In a first step, an initial search on Medline was undertaken to determine optimal keywords and include possible changes in the databases. The keywords used were double-checked before starting the main search. In a second step all identified keywords were used to search all the databases mentioned above. A third search was undertaken on the bibliographies of identified articles and previous meta-analyses. The initial keywords used were: Depressive Disorder NOT Depression, Postpartum NOT Seasonal Affective Disorder; Antidepressive Agents; Fluoxetine; Venlafaxine.

Unpublished studies were sought by communication with key researchers and key organizations (Food and Drug Administration and European Medicines Agency). A search on clinicaltrials.gov and Current Controlled Trial was also performed.

### Study selection

Eligibility assessment was performed independently in blinded standardized manner by 2 reviewers. Studies identified were grouped into two categories: RCTs and observational cohorts. Disagreements were resolved by consensus or in consultation with a third reviewer.

Studies appearing to duplicate authors, treatment comparisons, sample sizes and outcomes were checked one against another to avoid double-counting and integrating data from several reports on the same study.

### Assessment of Methodological Quality

Each paper was then assessed for methodological quality prior to inclusion in the review, using two appropriate standardized critical appraisal instruments [14], one for RCTs and one for observational studies (Appendix S1).

### Data Collection

A data extraction sheet based on the Cochrane Handbook for Systematic Reviews of Interventions guidelines Version 5.0.2 [15] was developed, pilot-tested on ten randomly-selected included studies, and refined accordingly. For each arm of the studies included, information was extracted on: 1/characteristics of the study (year, country, randomized or not? blinded or not? versus placebo or not? exclusion of patients on the basis of a placebo washout period or not? number of follow-up visits, number of arms, funding); 2/characteristics of trial participants (age, gender, number of patients included in analysis, type [inpatient (including studies with both inpatients and outpatients), outpatient and primary-care outpatient]); 3/type of intervention (treatment, dose, duration); 4/outcome measure (scale used, pre- and post-treatment mean and SD, type of analysis). Dosages were classified as “low”, “medium”, and “high” [16] (Table 1) or “variable”.

**Table 1:** Dosage classification.

	Low	Medium	High
**Fluoxetine**	<30 mg/day	30–50 mg/day	>50 mg/day
**Venlafaxine**	<153 mg/day	153–218,7 mg/day	>218,7 mg/day

One review author extracted these data from the studies included. The second author extracted the data from 10% of the studies to have an idea of the inter-rater reliability, and checked the data in the remaining studies. Authors of reviewed articles were contacted for further information and were asked for missing data when it was needed.

### Data analysis

The main criterion was the pre-/post-treatment difference on the depression scale in each study arm involving venlafaxine or fluoxetine or placebo. Taking into account the numerous criticisms on the use of effect sizes in meta-analyses [17], [18], we standardised the different instruments (mean and SD) by multiplying the scores by 100 and dividing them by the difference between the maximum possible value minus the minimum possible value, so that standardised scores range from 0 to 100. Then we calculated the raw mean difference D on these standardized scores. When D was reported in papers without corresponding estimates of variance, this variance was calculated from pre- and post-treatment variances when possible (using a pre-post- correlation estimated from the other studies). Heterogeneity between comparable studies was assessed using the Q statistic [19]. Publication bias was investigated graphically using funnel plots.

To adjust our comparison of observational studies and randomized controlled trials on identified sources of heterogeneity, and to quantify the impact of methodological choices on response, a meta-regression was performed. The dependent variable was D and the following explanatory variables were pre-specified: type of treatment (fluoxetine, venlafaxine, placebo); year of publication; depression scale used (HRSD-17, HRSD-21, MADRS); study duration; randomisation (yes/no); placebo design (yes/no); number of assessments; exclusion of placebo responders (yes/no); age; gender; patient type (outpatients in primary care/outpatients/inpatients); type of analysis (per protocol/intention to treat with last observation carried forward); baseline severity. This meta-regression was performed with the “study” factor specified as a random effect (mixed model). Studies were weighted by the inverse of D variance (n/var). Multiple imputation of missing data was performed using a Gibbs sampler [20].

To assess the robustness of our results, sensitivity analyses were performed: 1) using a wide range of correlation coefficients between pre- and post-treatment mean scores on the scale, 2) by removing each study in turn and 3) using the quality assessment to adjust the weight of a given study.

Analyses were performed using R (R Development Core Team) and the libraries meta (Schwarzer G), lme4 (Maechler D), and MICE (Van Buuren S, Groothuis-Oudshoorn K). Results are presented according to PRISMA statements [21] and MOOSE statements [22].

## Results

### Study selection

**Figure 1:**
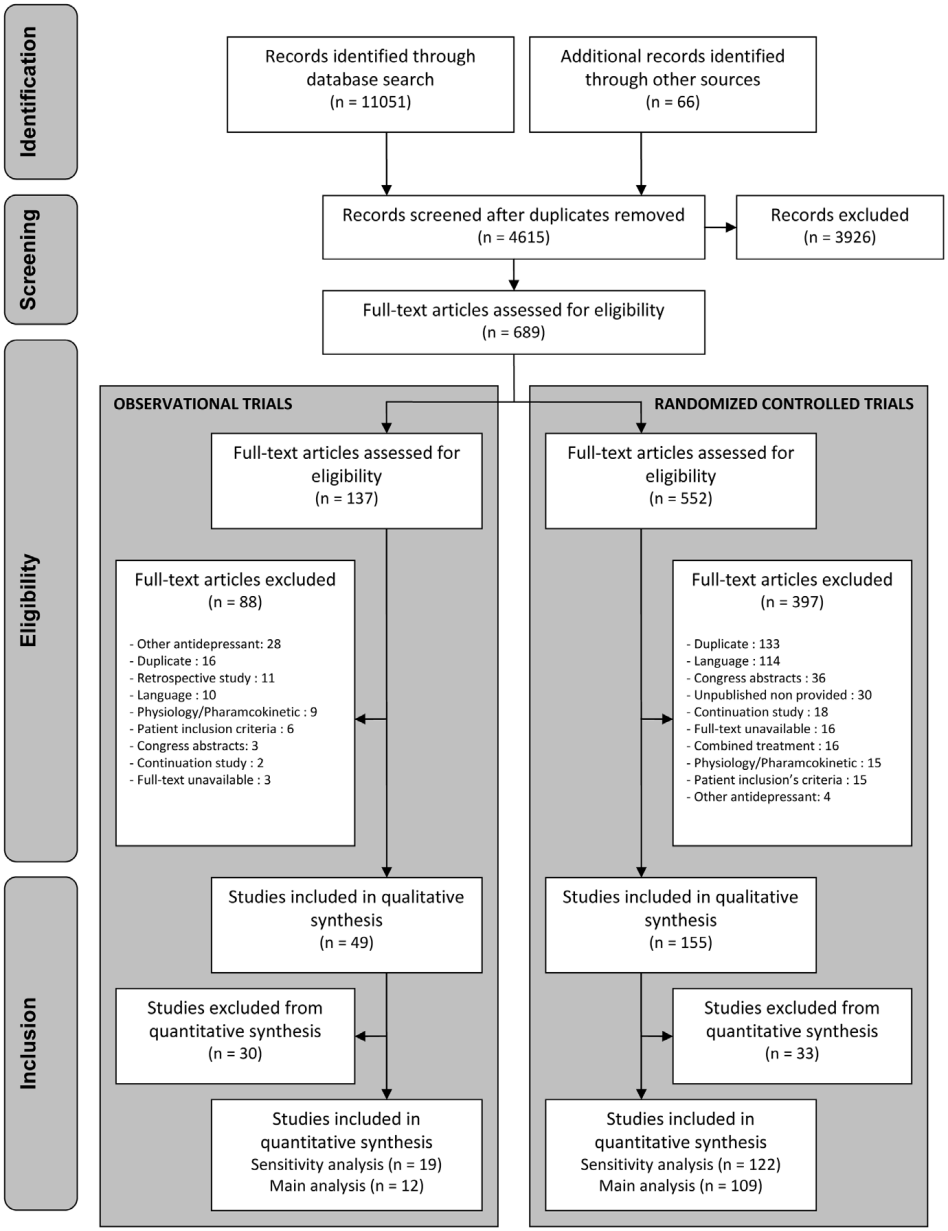
Flow Diagram.

The search of Medline, Cochrane and Embase databases provided a total of 11051 citations with respectively 2985, 3823 and 4243 citations. An additional 66 studies were identified by manual search. After adjusting for duplicates, 4615 remained. Of these, 3926 studies were discarded because, after review of the abstracts, it appeared that these papers did not meet the criteria. Of 33 unpublished relevant studies identified, only 3 were provided by pharmaceutical firms. 204 studies were included in the qualitative review and 141 in the quantitative review (covering the wider range of depressive disorders) with 121 studies in the main analysis. A flow chart detailing the study selection process for RCTs and observational studies is given in Figure 1.

### Study characteristics and risk of bias within studies

In the main analysis, the studies selected were 12 observational studies and 109 randomized controlled trials involving respectively 6757 and 11035 patients in 12 and 149 arms. In the depressive disorder spectrum analysis, the studies selected were 19 observational studies and 122 randomized controlled trials involving respectively 15753 and 12405 patients in 19 and 168 arms. A summary of study methodology, participants, intervention and quality is given in Table 2 and study characteristics are presented as a table in a web appendix (Appendix S1).

**Table 2:** Study description.

	Main analysis (Major depressive disorder)				Second analysis (depressive disorder spectrum)			
	Randomised Controlled Trials		Observational Trials		Randomised Controlled Trials		Observational Trials	
**Study methodology**								
Number of studies	109		12		122		19	
Year (Min-Max)	1989–2009		1994–2007		1989–2009		1994–2007	
Continent	(NA = 1)				(NA = 2)			
North America (%)	29	(26.8)	5	(41.7)	33	(27.5)	8	(42.1)
Central America and South America (%)	10	(9.3)	2	(16.7)	11	(9.2)	2	(10.5)
Europe (%)	48	(44.4)	4	(33,3)	55	(45.8)	8	(42.1)
Asia and Oceania (%)	10	(9.3)	1	(8.3)	10	(8.3)	1	(5.3)
Africa (%)	2	(1.9)	.		2	(1.7)	.	
Multi-continent (%)	9	(8.3)	.		9	(7.5)	.	
Blinded								
Yes (%)	100	(91.7)	.		111	(91.0)	.	
No (%)	9	(8.3)	12	(100)	11	(9.0)	19	(100)
Placebo design								
Yes (%)	22	(20.2)	.		26	(21.3)	.	
No (%)	87	(79.8)	12	(100)	96	(78.7)	19	(100)
Exclusion of placebo responders	(NA = 10)		(NA = 3)		(NA = 11)		(NA = 3)	
Yes (%)	60	(60.6)	1	(11,1)	67	(60.4)	3	(18.7)
No (%)	39	(39.4)	8	(88,9)	44	(39.6)	13	(81.3)
Number of follow-up visits (Min, Q1, median, Q2, Max)	(NA = 2)2, 5, 6, 7, 13		(NA = 1)2, 4, 5, 7, 10		(NA = 2)2, 5, 6, 7, 13		(NA = 1)2, 4, 5, 7, 14	
Study duration (Min, Q1, median, Q2, Max)	4, 6, 6, 8, 26		4, 8, 8, 17.25, 24		4, 6, 6, 8, 26		4, 8, 8, 20, 24	
Quality assessment/100 points (Min, Q1, median, Q2, Max)	57, 77, 80, 83, 100		54, 58, 62, 71, 75		57, 79, 80, 83, 100		54, 58, 62, 69, 75	
Funding	(NA = 36)		(NA = 1)		(NA = 43)		(NA = 4)	
Industry (%)	65	(89.0)	6	(54,5)	69	(87.4)	9	(60)
Mixt (public and industry) (%)	3	(4.1)	3	(27,3)	4	(5.0)	3	(40)
Public (%)	5	(6.9)	2	(18.2)	6	(7.6)	3	(40)
Analysis	(NA = 4)		(NA = 3)		(NA = 5)		(NA = 6)	
ITT with LOCF (%)	72	(68.6)	7	(77.8)	82	(70.1)	10	(76.9)
Per Protocol (%)	33	(31.4)	2	(22,2)	35	(29.9)	3	(23.1)
**Arm characteristics**								
Number of arms	149		12		168		19	
Treatment								
Fluoxetine (%)	80	(53,7)	5	(41.7)	92	(54.8)	7	(36.9)
Venlafaxine (%)	47	(31.5)	7	(58.3)	50	(29.8)	12	(63.1)
Placebo (%)	22	(14.8)	.		26	(15.4)	.	
Dose (Active treatment arms)	(NA = 22)				(NA = 26)			
Low (%)	47	(37.0)	5	(41.7)	52	(36.6)	7	(36.8)
Medium (%)	7	(5.5)	.		7	(4.9)	.	
High (%)	8	(6.3)	.		8	(5.7)	1	(5.3)
Variable (%)	65	(51,2)	7	(58.3)	75	(52.8)	11	(57.9)
Size (Min, Q1, median, Q2, Max)	10, 37, 62, 95, 320		62, 87.5, 119.5, 395.8, 4320		10, 38.75, 64, 95, 320		14, 70, 96, 407.5, 6719	
Patient type	(NA = 12)				(NA = 12)		(NA = 1)	
Inpatient (%)	33	(24.1)	1	(8.3)	34	(21.8)	1	(5.6)
Outpatient (%)	93	(67.9)	10	(83.4)	109	(69.9)	15	(83.3)
Primary Care (%)	11	(8)	1	(8.3)	13	(8.3)	2	(11.1)
**Characteristics of trial participants**								
Number of patients entering analysis	11035		6757		12405		15753	
Age								
Mean	(NA = 8) 42.5		46.9		(NA = 9) 42.5		47.9	
SD	(NA = 39) 7.4		(NA = 1) 6.9		(NA = 46) 7.4		(NA = 1) 7	
Proportion of women (%)	(NA = 7) 67.8		68.7		(NA = 7) 66.9		70.1	
Baseline severity (% of the scale)								
Mean	(NA = 2) 45.0		42.3		(NA = 2) 44.1		41.9	
SD	(NA = 39) 7.8		(NA = 1) 9.5		(NA = 43) 7.9		(NA = 1) 9.4	

Quality score is computed out of 100 points from the two Joanna Brigs Institute instruments.

Data shown here as NA (Non Available or Missing data) are imputed in the meta-regression models.

ITT with LOCF: Intention To Treat with Last Observation Carried Forward.

From 76 letters requesting information sent to authors, we were able to collect information about missing data for 13 studies.

### Results from individual studies and synthesis of results

As expected, using the Q statistic, significant heterogeneity was detected (p<0.0001) for: 1/active treatment effect in RCTs, 2/placebo effect in RCTs and 3/active treatment effect in observational studies. The Forest plot presenting individual study results is presented in the web appendix (figure S1). Multivariate meta-regression (Table 3) showed that RCTs overestimate the standardised treatment response by a magnitude of 4.59 ([95% confidence interval] 2.61 to 6.56) in the main analysis and by 2.45 (0.97 to 3.93) in the depressive disorder spectrum analysis. In the main analysis, certain study design factors were associated with substantial variations in the standardised treatment response. The increase in treatment response was 0.27 (0.14 to 0.40) for each additional week of duration, 0.33 (0.11 to 0.55) for each additional follow-up assessment and 0.07 (0.01 to 0.13) for each year of study publication. In studies involving outpatients and in those with outpatients in primary care, patient improvement was respectively 1.81 (0.88 to 2.72) and 3.73 (2.37 to 5.09) greater than improvement observed in studies involving inpatients. Overestimated treatment response attributable to per-protocol analysis was 2.52 (1.45 to 3.6) when compared to intention-to-treat analysis. The standardised treatment response was smaller in double-blind studies than in open-label studies by a magnitude of 5.21 (−6.85 to −3.57). Similarly, when there was a placebo arm, treatment response was smaller by 4.54 (−5.50 to −3.58). Regarding patients, the standardised treatment response increased with mean baseline severity by 0.78 (0.71 to 0.84) for each percentage value of the severity scale that was used, and decreased by 0.16 (−0.26 to −0.07) for an increase of 1 year in the mean age of patients. The standardized treatment response for a placebo was 3.35 (−3.97 to −2.74) less than the response for fluoxetine, while the treatment response for venlafaxine was greater than that for fluoxetine by 2.51 (1.88 to 3.14).

**Table 3:** Meta-regression analysis.

	Main analysis		Depressive disorders spectrum analysis	
	Coefficient	[95% confidence interval]	Coefficient	[95% confidence interval]
**Arm characteristics**				
RCT (Ref = Observational)	4.59	[2.61 to 6.56]	2.45	[0.97 to 3.93]
Scale (Ref = HAM-21)				
HAMD-17	−2.32	[−3.03 to −1.61]	−1.66	[−2.29 to −1.03]
MADRS	−1.26	[−2.62 to 0.09]	−1.34	[−2.10 to 0.57]
Treatment (Ref = Fluoxetine)				
Placebo	−3.35	[−3.97 to −2.74]	−3.42	[−3.93 to −2.92]
Venlafaxine	2.51	[1.88 to 3.14]	2.25	[1.81 to 2.70]
Double blind study (Ref = No)	−5.21	[−6.85 to −3.57]	−3.37	[−4.65 to −2.09]
Placebo design study (Ref = No)	−4.54	[−5.50 to −3.58]	−3.54	[−4.21 to −2.86]
Year of publication	0.07	[0.01 to 0.13]	0.10	[0.05 to 0.15]
Duration	0.27	[0.14 to 0.40]	0.17	[0.11 to 0.23]
Number of follow up assessments	0.33	[0.11 to 0.55]	0.26	[0.13 to 0.39]
Exclusion of placebo responders	−0.27	[−1.06 to 0.52]	−0.08	[−0.71 to 0.54]
Type of analysis PP (Ref = ITT with LOCF)	2.52	[1.45 to 3.6]	2.55	[1.87 to 3.23]
Patient type (Ref = Inpatients)				
Outpatients	1.81	[0.88 to 2.72]	2.81	[2.18 to 3.43]
Outpatients in primary care	3.73	[2.37 to 5.09]	3.69	[2.59 to 4.80]
**Patient characteristics**				
Mean age	−0.16	[−0.26 to −0.07]	−0.05	[−0.11 to 0.01]
Gender	0.00	[−0.03 to 0.04]	0.01	[−0.02 to 0.04]
Baseline severity	0.78	[0.71 to 0.84]	0.83	[0.78 to 0.88]

Results are expressed in points of the standardised difference in mean.

Ref: reference.

PP: Per Protocol.

ITT with LOCF: Intention To Treat with Last Observation Carried Forward.

Results of the depressive disorders spectrum analysis show the robustness of our model (Table 3).

To assess the validity of our model we checked that the Variance Inflation Factor values were under 10 (all were under 3) and we checked that the normality of the residues was verified.

### Risk of bias across studies

**Figure 2:**
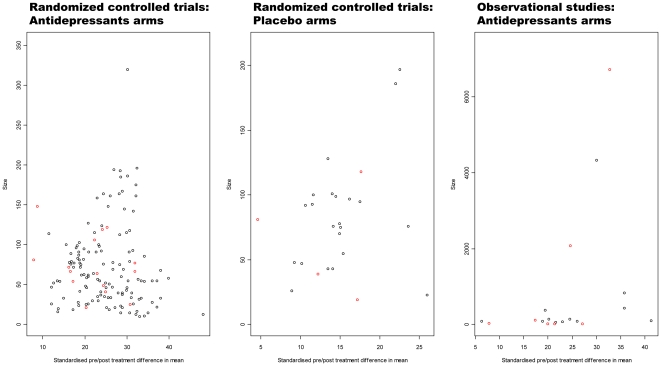
Funnel plots are presented for all types of arms: the left-hand plot concerns antidepressant arms in randomized controlled trials (RCTs), the middle plot concerns placebo arms in RCTs and the right-hand plot concerns antidepressant arms in observational studies. For each arm, the x-axis presents the standardised pre/post treatment difference in mean and the y-axis presents the number of patients analysed. The black dots represent studies in the main analysis and red dots studies added in the depressive disorders spectrum analysis.

Three funnel plots were drawn (Figure 2) for antidepressants in randomized controlled trials, antidepressants in observational studies and placebo in randomized controlled trials. The antidepressant arms in RCTs and in observational studies did not show evidence of any marked asymmetry whereas the funnel plot investigating placebo arms in randomized controlled trial shows some asymmetry.

### Additional analysis

Sensitivity analyses using various pre-post treatment correlation coefficients and taking quality into account showed the robustness of our estimations.

Sensitivity analysis, removing each study one at a time, identified a potential outlier among the observational studies [23]. This is a four-week observational study on fluoxetine in which the treatment effect is small (3.3 points on the HAMD-17). When it was removed, the coefficient representing the difference between randomized controlled trials and observational studies decreased from 4.59 to 1.67, remaining statistically significant in the main analysis, and decreased from 2.45 to 1.04 in the depressive disorder spectrum analysis.

Another potential outlier [24] was noticed among the studies included in the depressive disorders spectrum analysis. Once it was removed, the coefficients increased from 2.45 to 4.49. It is remarkable that this last value is close to the coefficient of 4.59 obtained in the main analysis. This study is in fact a randomized controlled study designed to measure effectiveness, which is not unlike an observational study.

We also performed: 1/a *post hoc* analysis (without placebo arms) adding the variable treatment dosage to assess its impact; it did not prove to be statistically significant, whereas estimations of other coefficients were unchanged; 2/a *post hoc* analysis, excluding three studies suspected of multiple publications, and we found no difference in our results; 3/a *post hoc* analysis using the standard deviation of the pre/post treatment difference as a standardization unit (i.e. (Post score-Pre score)/SD of the score difference) which produced similar results.

## Discussion

### Summary of evidence

Our results highlight a difference in patient response to treatment between RCTs and observational studies with a larger estimate in RCTs.

Certain design factors associated with treatment response, already reported in the literature, were here evidenced in the context of a study including both RCTs and observational studies. This can be considered as an element of external validity as regards the present study. Treatment and placebo mean responses increase with depression severity [2], [3], with duration of treatment, with the number of follow up assessments [5] and with the year of publication of the study [4]. Per-protocol analysis gives larger response estimates when compared to intention to treat analysis. We also found that mean antidepressant response decreases with placebo design and with blinded design. This could have a relationship with patient expectations for treatment effect [6], or rather with clinician expectations, since the depression scales considered here were clinician-version evaluations. Independently from baseline severity, inpatient mean treatment response is smaller compared to primary care patients or outpatients. This could relate to the higher levels of psychiatric comorbidities presented by inpatients. Venlafaxine and fluoxetine obtain a better response than placebo, and venlafaxine a better response than fluoxetine [16] even if it is small [25].

### Perspective

Randomized controlled trials are considered as the gold standard in the hierarchy of research designs for evaluating the efficacy and safety of a treatment intervention. Two major benefits are expected from randomization [26]: unbiased allocation of treatment, and application of statistical theory on the basis of random sampling which makes it possible to infer the specific treatment effect, especially when there is a blinded allocation of treatments. Another important argument in favor of RCTs can be derived from their methodological characteristics: they are so well documented and they rely on so simple a statistical paradigm that they can resist the major financial conflicts of interest inherent in the evaluation of pharmaceuticals. It can be recalled that the global pharmaceuticals market represents about 1% of the world gross domestic product [27].

Nevertheless, RCTs are criticised. First they are expensive, and indeed increasingly so. This reduces their feasibility and has potential consequences on the prices of new medication which are likely to become incompatible with the restrictions in health care resources. Second, RCTs raise ethical considerations, especially in the field of antidepressants where trials are still performed against a placebo, although older antidepressants are widely considered to be the best control alternative [28]. Even if such studies are justified, approved by ethics committees and required by regulatory agencies, at patient or investigator level the design can limit inclusion of patients needing active treatment. Thirdly, in the field of antidepressants, the ability of a double-blind design to preserve the benefit of randomisation is disputed [29]. Finally, they do not closely reflect clinical practice and lack of external validity [9], [30].

Observational studies have better external validity and they have other characteristics that make them useful sources of evidence, in that they tend to last longer and to enrol more patients than do randomized trials [31]. Statistical modelling should enable adjustment on known [32] or any potential [33] confounding factors, thus increasing the internal validity of such studies.

Only two naturalistic studies, both in a post hoc analysis, have explored the question of the link between antidepressant efficacy and effectiveness. In a retrospective analysis of a cohort of 1,014 inpatients [34], patients eligible (on the basis of classic inclusion criteria) for a RCT and patients not eligible differed significantly on several baseline measures and final Global Assessment of Functioning scores but not on any other outcome measures such as depression rating scales. However, this study only investigated inpatients (a more homogenous population) and the analysis was not adjusted on prognosis factors at baseline or on treatment associated (psychotherapy, use of a pharmacological augmentation strategy).

In another similar analysis applied to the outpatient STAR-D cohort [10], the authors found that patients eligible for a RCT had a better response which persisted even after adjustments for baseline differences. The design of this study is more efficient in controlling for confounders such as psychotherapy and pharmacological augmentation strategies.

Thus the place of observational studies in treatment effect assessment is open to discussion. Our study is of interest in this debate because we found a difference in response to antidepressants between the two approaches, with larger estimates in RCTs than in observational studies. However, this difference does not constitute a clear clinical difference. The National Institute for Clinical Excellence has suggested that at least a 3-point difference is needed on the Hamilton scale to claim a clinically significant effect [35]. This corresponds to a variation of 5.8 points on our standardized score, whereas the adjusted mean difference here is 4.59. Nevertheless the small clinical relevance of this difference should be put in perspective, and it is remarkable that it is very similar to the difference between the antidepressant and placebo responses estimated in the present study. Thus, being a little bit provocative, two points of view are possible: 1/if one believes that antidepressants have greater effect than placebos, then there is indeed a large difference between treatment response as estimated by observational studies and treatment effect estimated by RCTs; 2/if one considers that antidepressants are not actually more efficient than placebos [36] the difference between observational studies and randomized controlled trial can also be considered as small, but no longer relevant.

Furthermore, these two thresholds for clinical significance (the NICE threshold and the difference between placebo and antidepressant) can be used to interpret all coefficients estimated with our model.

### Limitations

The limitations of a meta-analysis are linked to the limitations of the individual studies included [37]. As we used observational trials and the arms of the RCTs were separated, our work has a level of evidence coherent with observational study meta-analysis. Since confounders could be present when comparing treatment effect in observational studies and in RCTs, we used a meta-regression. However this approach can also present limitations [38]. It is more likely to detect effects at study level, but it can lead to misinterpretations at patient level, where an aggregation bias can occur, and this cannot be investigated without individual patient data [39]. Thus results relating to patient characteristics such as gender, severity, and age should be interpreted cautiously.

Our choice of a standardisation of pre/post treatment scores by multiplying the scores by 100 and dividing them by the possible range of the instrument could be criticised or at least appear as unconventional compared to the more classic use of a standard deviation of the pre/post treatment difference as a standardization unit (i.e. (Post score-Pre score)/SD of the score difference).

Nevertheless, we support our a priori choice for four principal reasons relating to our objective: 1/the standard deviation of the score difference (i.e. the variability) is not solely due to differences on the scale, it is also due to patient heterogeneity in the studies. This could lead mathematically to an underestimation of classic outcomes such as (Post score-Pre score)/(SD of the score difference) in observational studies (where there is great heterogeneity) as compared to RCTs. 2/it appears as the simplest statistic to interpret for clinicians (% of variation of a scale) 3/as we had to impute variance for several standard deviations of the score difference, the imputed data were not used for the calculation of our principal outcome. Indeed, in meta-analysis, multiple imputation is frequent for the variance of an outcome [40] but it could be problematic if it directly concerns the outcome 4/the use of effect size is criticised in the literature [17], [18].

Thus, taking the precaution to consider the depression scale used as an explanatory variable in our regression model, we are confident in this outcome. In addition, we performed a post hoc sensitivity analysis using a classic method to standardise scales. It led to the same conclusion (i.e. there is a larger estimate of treatment response in RCTs).

A publication bias, which could involve a differential between randomized controlled trials and observational studies, might account for some of the effect we observed. However, the funnel plots of antidepressant response suggest that selective reporting did not lead to an overestimation of D in RCTs or in observational studies. Conversely, in randomized controlled trials on antidepressants against placebo, the funnel plot of the placebo response is asymmetrical, which illustrates a known publication bias [1] with an underestimation of placebo response in these studies.

A four-week observational study in which the mean pre-post treatment difference is small [23] could have led to an overestimation of the difference between observational studies and RCTs. However it met our inclusion criteria and there is basically no reason to remove it. One could object that so short a study duration is not sufficient for an observational study. In fact, the small effect makes sense from a clinical point of view, because one month is typically the time lapse clinicians choose to discontinue an ineffective treatment [41].

### Conclusions

#### Implications for practice

In their day-to-day practice, clinicians and health authorities generally evaluate the effectiveness of new medication from RCTs. In the field of antidepressants it should be known that, as already demonstrated on non-antidepressant drug studies [42], larger efficacy estimates can be expected in optimal experimental conditions than the effectiveness estimates obtained in real-word setting. This could have implications as to which patients should be treated by the clinician, and what costs the health authority should cover.

#### Implications for research

Observational studies should be considered as a necessary complement to randomized controlled trials. Phase IV studies should not be restricted to the study of the safety of a product, they should also study effectiveness.

Describing the design factors that can modify measures of antidepressant response will help researchers to choose more appropriate designs and to find a balance between internal and external validity. This has ethical implications because patient improvement is linked to the design. Our work could help to draft guidelines defining what design antidepressant efficacy and effectiveness trials should adopt.

## Supporting Information

Figure S1 **Forest plots are presented for all types of arms for the main analysis.** Studies on the wider spectrum of depressive disorders are not presented here for legibility. Confidence intervals are given with only one imputation, for descriptive purposes. Because of a great heterogeneity, summary measures are not given.
(TIF)

Appendix S1 **Web Appendix.**
(DOC)
