# Supplementary figures and images for: Antidepressant Response in Major Depressive Disorder: A Meta-Regression Comparison of Randomized Controlled Trials and Observational Studies
Source: PLoS One. 2011 Jun 8;6(6):e20811. doi: 10.1371/journal.pone.0020811 (PMC3110792; doi:10.1371/journal.pone.0020811)

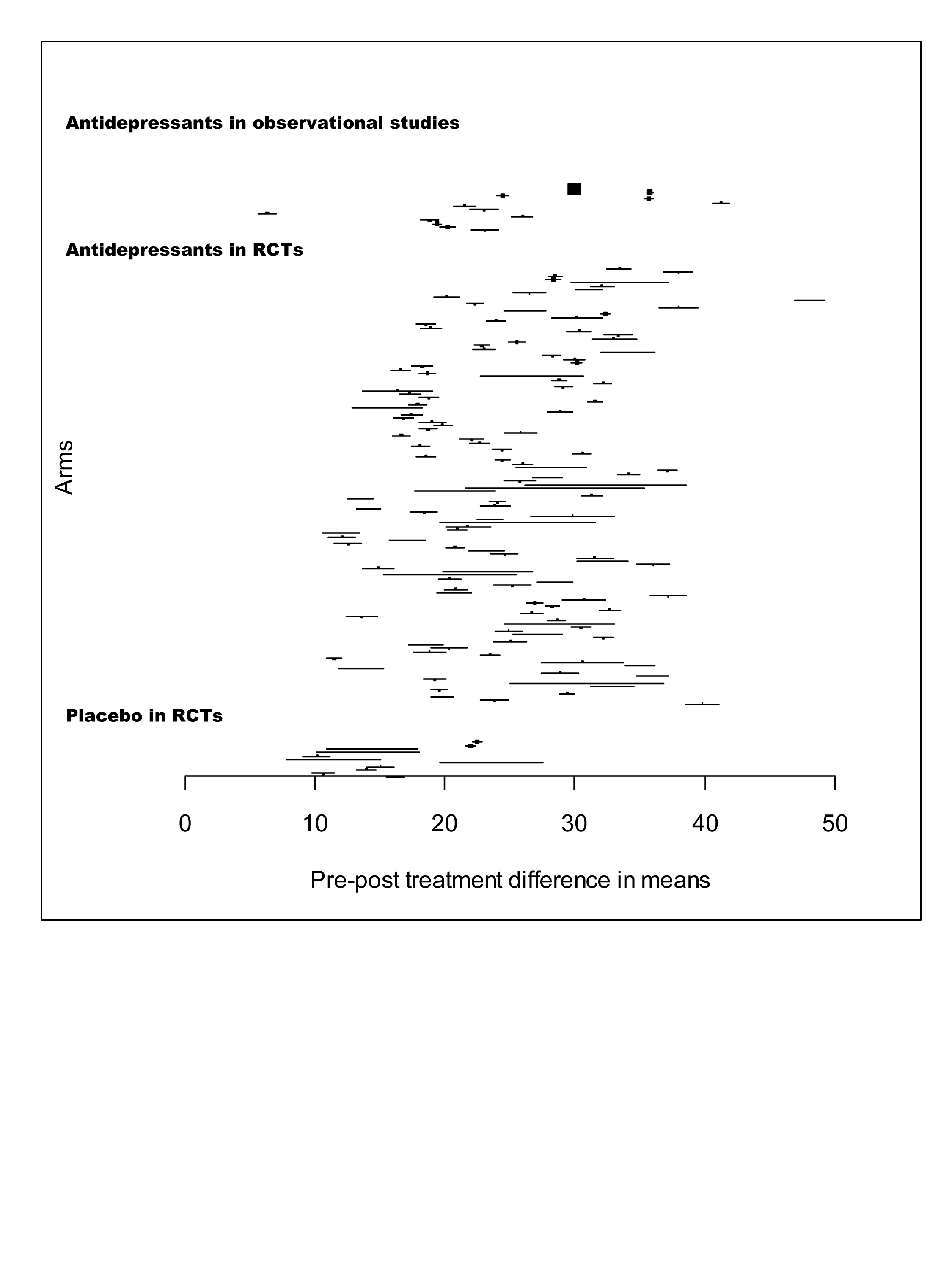

Supplement: Figure S1 — Forest plots are presented for all types of arms for the main analysis. Studies on the wider spectrum of depressive disorders are not presented here for legibility. Confidence intervals are given with only one imputation, for descriptive purposes. Because of a great heterogeneity, summary measures are not given. (TIF) [file pone.0020811.s001.tif]
