# Supplementary material for: Antidepressant Response in Major Depressive Disorder: A Meta-Regression Comparison of Randomized Controlled Trials and Observational Studies
Source: PLoS One. 2011 Jun 8;6(6):e20811. doi: 10.1371/journal.pone.0020811 (PMC3110792; doi:10.1371/journal.pone.0020811)
Supplement: Appendix S1 — Web Appendix. (DOC) [file pone.0020811.s002.doc]

**Web Appendix**

Randomized Controlled Trials

| Author | Year | Analysis | Was the assignment to treatment groups truly random? | Were participants blinded to treatment allocation? | Was allocation to treatment groups concealed from the allocator? | Were the outcomes of people who withdrew described and included in the analysis? | Were those assessing outcomes blind to the treatment allocation? | Were the control and treatment groups comparable at entry? | Were groups treated identically other than for the named interventions? | Were outcomes measured in the same way for all groups? | Were outcomes measured in a reliable way? | Was appropriate statistical analysis used? |
| --- | --- | --- | --- | --- | --- | --- | --- | --- | --- | --- | --- | --- |
| Aguglia | 1993 | Main | 😐 | ☺ | ☺ | ☹ | ☺ | ☹ | 😐 | ☺ | ☺ | ☹ |
| AK130940 | 2005 | Main | 😐 | ☺ | ☺ | ☹ | ☺ | 😐 | 😐 | ☺ | ☺ | ☺ |
| Akhondzadeh | 2007 | Main | ☺ | ☺ | ☺ | ☹ | ☺ | ☺ | 😐 | ☺ | ☺ | ☹ |
| Akhondzadeh | 2003 | Main | 😐 | ☺ | ☺ | ☹ | ☺ | 😐 | 😐 | ☺ | ☺ | ☹ |
| Akkaya | 2006 | Main | 😐 | ☹ | ☹ | 😐 | ☹ | 😐 | 😐 | ☺ | ☺ | ☹ |
| Alves | 1999 | Main | ☺ | ☺ | ☺ | 😐 | ☺ | ☺ | 😐 | ☺ | ☺ | ☺ |
| Amini | 2005 | Main | 😐 | ☺ | ☺ | ☹ | ☺ | ☺ | 😐 | ☺ | ☺ | ☹ |
| Amsterdam | 1998 | Main | 😐 | ☺ | ☺ | ☹ | ☺ | ☺ | 😐 | ☺ | ☺ | ☹ |
| Andreoli | 2002 | Main | 😐 | ☺ | ☺ | ☹ | ☺ | ☺ | ☺ | ☺ | ☺ | ☺ |
| Ansseau | 1994 | Main | 😐 | ☺ | ☺ | ☹ | ☺ | ☺ | ☺ | ☺ | ☺ | ☺ |
| Badyal | 2006 | Main | ☺ | ☹ | ☹ | 😐 | ☹ | ☺ | 😐 | ☺ | ☺ | ☹ |
| Beasley | 1993 | Main | 😐 | ☺ | ☺ | ☹ | ☺ | ☺ | 😐 | ☺ | ☺ | ☹ |
| Behnke | 2002 | Main | 😐 | ☺ | ☺ | ☹ | ☺ | ☺ | 😐 | ☺ | ☺ | ☹ |
| Benkert | 2006 | Main | 😐 | ☺ | ☺ | 😐 | ☺ | 😐 | 😐 | ☺ | ☺ | ☺ |
| Bennie | 1995 | Main | 😐 | ☺ | ☺ | ☹ | ☺ | ☺ | ☺ | ☺ | ☺ | ☹ |
| Berlanga | 1997 | Main | 😐 | ☺ | ☺ | ☹ | ☺ | ☺ | 😐 | ☺ | ☺ | ☹ |
| Besançon | 1993 | Main | 😐 | ☺ | ☺ | ☹ | ☺ | 😐 | 😐 | ☺ | ☺ | ☹ |
| Bjerkenstedt | 2004 | Main | 😐 | ☺ | ☺ | ☹ | ☺ | ☺ | 😐 | ☺ | ☺ | ☹ |
| Bowden | 1993 | Main | 😐 | ☺ | ☺ | ☹ | ☺ | ☺ | 😐 | ☺ | ☺ | ☹ |
| Boyer | 1998 | Main | 😐 | ☺ | ☺ | ☹ | ☺ | ☺ | 😐 | ☺ | ☺ | ☹ |
| Chouinard | 1999 | Main | 😐 | ☺ | ☺ | ☹ | ☺ | ☺ | 😐 | ☺ | ☺ | ☹ |
| Clerc | 1994 | Main | 😐 | ☺ | ☺ | 😐 | ☺ | ☺ | 😐 | ☺ | ☺ | ☹ |
| Corne | 1989 | Main | 😐 | ☺ | ☺ | ☹ | ☺ | ☺ | 😐 | ☺ | ☺ | ☹ |
| Corrigan | 2000 | Main | 😐 | ☺ | ☺ | ☹ | ☺ | 😐 | 😐 | ☺ | ☺ | ☹ |
| Costa E Silva | 1998 | Main | 😐 | ☺ | ☺ | 😐 | ☺ | ☺ | 😐 | ☺ | ☺ | ☹ |
| Cunningham | 1997 | Main | 😐 | ☺ | ☺ | ☹ | ☺ | ☺ | 😐 | ☺ | ☺ | ☹ |
| Cunningham | 1994 | Main | 😐 | ☺ | ☺ | ☹ | ☺ | ☺ | 😐 | ☺ | ☺ | ☹ |
| Dalery | 1992 | Main | 😐 | ☺ | ☺ | ☹ | ☺ | ☺ | 😐 | ☺ | ☺ | ☹ |
| Dalery | 2003 | Main | 😐 | ☺ | ☺ | ☹ | ☺ | 😐 | 😐 | ☺ | ☺ | ☹ |
| De Jonghe | 1991 | Main | 😐 | ☺ | ☺ | ☺ | ☺ | 😐 | ☺ | ☺ | ☺ | ☹ |
| De_Wilde | 1993 | Main | 😐 | ☺ | ☺ | ☹ | ☺ | ☺ | 😐 | ☺ | ☺ | ☹ |
| Diaz_Martinez | 1998 | Main | 😐 | ☹ | ☹ | ☹ | ☹ | ☺ | 😐 | ☺ | ☺ | ☹ |
| Dichter | 2005 | Main | 😐 | ☹ | 😐 | ☹ | ☺ | 😐 | 😐 | ☺ | ☺ | ☹ |
| Dierick | 1996 | Main | 😐 | ☺ | ☺ | ☹ | ☺ | ☺ | ☺ | ☺ | ☺ | ☹ |
| Fava | 2005 | Main | 😐 | ☺ | ☺ | ☹ | ☺ | 😐 | 😐 | ☺ | ☺ | ☹ |
| Fawcett | 1989 | Main | 😐 | ☺ | ☺ | ☹ | ☺ | 😐 | 😐 | ☺ | ☺ | ☹ |
| Feighner | 1989 | Main | 😐 | ☺ | ☺ | ☹ | ☺ | 😐 | 😐 | ☺ | ☺ | ☹ |
| Feighner | 1991 | Main | 😐 | ☺ | ☺ | ☹ | ☺ | 😐 | 😐 | ☺ | ☺ | ☹ |
| Ferreri | 1989 | Main | 😐 | ☺ | ☺ | ☹ | ☺ | ☺ | 😐 | ☺ | ☺ | ☹ |
| Gagiano | 1993 | Main | 😐 | ☺ | ☺ | ☹ | ☺ | ☺ | 😐 | ☺ | ☺ | ☹ |
| Gattaz | 1995 | Main | 😐 | ☺ | ☺ | ☹ | ☺ | 😐 | 😐 | ☺ | ☺ | ☹ |
| Geerts | 1994 | Main | ☺ | ☺ | ☺ | ☹ | ☺ | ☹ | 😐 | ☺ | ☺ | ☹ |
| Gentil | 2000 | Main | 😐 | ☺ | ☺ | ☹ | ☺ | ☺ | ☹ | ☺ | ☺ | ☹ |
| Ginestet | 1989 | Main | 😐 | ☺ | ☺ | ☹ | ☺ | ☺ | 😐 | ☺ | ☺ | ☹ |
| Goldstein | 2002 | Main | 😐 | ☺ | ☺ | ☺ | ☺ | ☺ | 😐 | ☺ | ☺ | ☺ |
| Gorenstein | 2002 | Main | 😐 | ☺ | ☺ | ☹ | ☺ | 😐 | 😐 | ☺ | ☺ | ☹ |
| Guelfi | 2000 | Main | ☺ | ☺ | ☺ | ☹ | ☺ | ☺ | ☺ | ☺ | ☺ | ☺ |
| Guelfi | 1995 | Main | 😐 | ☺ | ☺ | ☹ | ☺ | ☺ | ☺ | ☺ | ☺ | ☹ |
| Guelfi | 1998 | Main | 😐 | ☺ | ☺ | ☹ | ☺ | ☺ | 😐 | ☺ | ☺ | ☹ |
| Heiligenstein | 1993 | Main | 😐 | ☺ | ☺ | ☹ | ☺ | ☹ | 😐 | ☺ | ☺ | ☹ |
| Hong | 2003 | Main | 😐 | ☺ | ☺ | ☹ | ☺ | ☺ | 😐 | ☺ | ☺ | ☹ |
| Joyce | 2002 | Main | 😐 | ☹ | ☹ | ☹ | ☹ | 😐 | 😐 | ☺ | ☺ | ☹ |
| Judd | 1993 | Main | 😐 | ☹ | ☹ | ☹ | ☹ | 😐 | 😐 | ☺ | ☺ | ☹ |
| Kennedy | 2008 | Main | 😐 | ☺ | ☺ | ☺ | ☺ | ☺ | 😐 | ☺ | ☺ | ☹ |
| Khan | 1998 | Main | 😐 | ☺ | ☺ | ☹ | ☺ | ☺ | 😐 | ☺ | ☺ | ☹ |
| Kuha | 1991 | Main | 😐 | ☺ | ☺ | ☹ | ☺ | ☺ | ☺ | ☺ | ☺ | ☹ |
| Lapierre | 1997 | Main | 😐 | ☺ | ☺ | ☹ | ☺ | ☺ | 😐 | ☺ | ☺ | ☹ |
| Lecrubier | 1997 | Main | 😐 | ☺ | ☺ | ☹ | ☺ | ☺ | 😐 | ☺ | ☺ | ☹ |
| Lee | 2005 | Main | 😐 | ☺ | ☺ | ☹ | ☺ | ☺ | ☺ | ☺ | ☺ | ☹ |
| Lemoine | 2007 | Main | ☺ | ☺ | ☺ | ☺ | ☺ | ☺ | ☺ | ☺ | ☺ | ☺ |
| Levine | 1989 | Main | 😐 | ☺ | ☺ | ☹ | ☺ | 😐 | 😐 | ☺ | ☺ | ☹ |
| Loeb | 1989 | Main | 😐 | ☺ | ☺ | ☹ | ☺ | 😐 | 😐 | ☺ | ☺ | ☹ |
| Loo | 1999 | Main | 😐 | ☺ | ☺ | ☹ | ☺ | ☺ | 😐 | ☺ | ☺ | ☺ |
| Mac_Partlin | 1998 | Main | 😐 | ☺ | ☺ | ☹ | ☺ | ☺ | 😐 | ☺ | ☺ | ☹ |
| Mao | 2008 | Main | 😐 | ☺ | ☺ | ☹ | ☺ | ☺ | 😐 | ☺ | ☺ | ☺ |
| Martenyi | 2001 | Main | 😐 | ☺ | ☺ | ☹ | ☺ | ☺ | 😐 | ☺ | ☺ | ☹ |
| Massana | 1998 | Main | 😐 | ☺ | ☺ | ☹ | ☺ | 😐 | 😐 | ☺ | ☺ | ☹ |
| Mehtonen | 2000 | Main | 😐 | ☺ | ☺ | ☹ | ☺ | ☺ | ☹ | ☺ | ☺ | 😐 |
| Mendels | 1993 | Main | 😐 | ☺ | ☺ | ☹ | ☺ | ☺ | 😐 | ☺ | ☺ | ☹ |
| Montgomery | 2004 | Main | 😐 | ☺ | ☺ | ☹ | ☺ | ☺ | 😐 | ☺ | ☺ | ☺ |
| Moreno | 2005 | Main | 😐 | ☺ | ☺ | ☹ | ☺ | ☹ | 😐 | ☺ | ☺ | ☺ |
| Nemeroff | 2007 | Main | 😐 | ☺ | ☺ | ☹ | ☺ | ☺ | 😐 | ☺ | ☺ | ☹ |
| Noguera | 1991 | Main | 😐 | ☺ | ☺ | ☹ | ☺ | 😐 | 😐 | ☺ | ☺ | ☹ |
| Noorbala | 2005 | Main | ☺ | ☺ | ☺ | ☹ | ☺ | 😐 | 😐 | ☺ | ☺ | ☺ |
| Novotny | 2002 | Main | 😐 | ☺ | ☺ | ☹ | ☺ | ☺ | 😐 | ☺ | ☺ | ☹ |
| Ontiveros | 1997 | Main | 😐 | ☺ | ☺ | ☹ | ☺ | ☺ | ☺ | ☺ | ☺ | ☹ |
| Ontiveros | 1998 | Main | 😐 | ☺ | ☺ | ☹ | ☺ | ☺ | 😐 | ☺ | ☺ | ☹ |
| Owens | 2008 | Main | ☺ | ☺ | ☺ | ☹ | ☺ | 😐 | 😐 | ☺ | ☺ | ☹ |
| Patris | 1996 | Main | 😐 | ☺ | ☺ | ☹ | ☺ | ☺ | ☺ | ☺ | ☺ | ☺ |
| Rapaport | 1996 | Main | 😐 | ☺ | ☺ | ☹ | ☺ | ☺ | 😐 | ☺ | ☺ | ☺ |
| Remick | 1993 | Main | 😐 | ☺ | ☺ | ☹ | ☺ | ☺ | 😐 | ☺ | ☺ | ☹ |
| Remick | 1989 | Main | 😐 | ☺ | ☺ | ☹ | ☺ | ☺ | 😐 | ☺ | ☺ | ☹ |
| Reynaert | 1994 | Main | ☺ | ☺ | ☺ | ☹ | ☺ | ☺ | ☺ | ☺ | ☺ | ☹ |
| Ropert | 1989 | Main | 😐 | ☺ | ☺ | ☹ | ☺ | ☺ | 😐 | ☺ | ☺ | ☹ |
| Rudolph | 1998 | Main | ☺ | ☺ | ☺ | ☹ | ☺ | ☺ | 😐 | ☺ | ☺ | ☹ |
| Rudolph | 1999 | Main | ☺ | ☺ | ☺ | ☹ | ☺ | ☺ | 😐 | ☺ | ☺ | ☺ |
| Samuelian | 1998 | Main | 😐 | ☺ | ☺ | ☹ | ☺ | ☺ | 😐 | ☺ | ☺ | ☹ |
| Sauer | 2003 | Main | 😐 | ☺ | ☺ | ☹ | ☺ | ☺ | 😐 | ☺ | ☺ | ☺ |
| Schrader | 2000 | Main | 😐 | ☺ | ☺ | ☹ | ☺ | ☺ | 😐 | ☺ | ☺ | ☺ |
| Schweizer | 1994 | Main | 😐 | ☺ | ☺ | ☹ | ☺ | ☺ | 😐 | ☺ | ☺ | ☹ |
| Sechter | 1999 | Main | 😐 | ☺ | ☺ | ☹ | ☺ | 😐 | 😐 | ☺ | ☺ | ☹ |
| Sheehan | 2009 | Main | ☺ | ☺ | ☺ | ☹ | ☺ | ☹ | 😐 | ☺ | ☺ | ☺ |
| Shelton | 2006 | Main | 😐 | ☺ | ☺ | ☹ | ☺ | ☺ | 😐 | ☺ | ☺ | ☺ |
| Sir | 2005 | Main | ☺ | ☺ | ☺ | ☹ | ☺ | ☺ | 😐 | ☺ | ☺ | ☺ |
| Suleman | 1997 | Main | 😐 | ☺ | ☺ | ☹ | ☺ | ☹ | 😐 | ☺ | ☺ | ☹ |
| Suri | 2000 | Main | 😐 | ☹ | ☹ | ☹ | ☺ | ☺ | ☹ | ☺ | ☺ | ☹ |
| Tamminen | 1989 | Main | 😐 | ☺ | ☺ | ☹ | ☺ | ☺ | ☺ | ☺ | ☺ | ☹ |
| Thase | 1997 | Main | 😐 | ☺ | ☺ | ☹ | ☹ | ☺ | 😐 | ☺ | ☺ | ☹ |
| Thase | 2006 | Main | 😐 | ☺ | ☺ | ☹ | ☹ | 😐 | 😐 | ☺ | ☺ | ☹ |
| Tignol | 1993 | Main | 😐 | ☺ | ☺ | ☹ | ☺ | ☺ | 😐 | ☺ | ☺ | ☹ |
| Tollefson | 1994 | Main | 😐 | ☺ | ☺ | ☹ | ☺ | ☺ | 😐 | ☺ | ☺ | ☹ |
| Tural | 2003 | Main | 😐 | ☹ | ☹ | ☹ | 😐 | ☺ | 😐 | ☺ | ☺ | ☹ |
| Tylee | 1997 | Main | ☺ | ☺ | ☺ | ☹ | ☺ | 😐 | 😐 | ☺ | ☺ | ☹ |
| Tzanakaki | 2000 | Main | ☹ | ☺ | ☺ | ☹ | ☺ | ☺ | ☺ | ☺ | ☺ | ☹ |
| Van_Moffaert | 1995 | Main | 😐 | ☺ | ☺ | ☹ | ☺ | ☺ | 😐 | ☺ | ☺ | ☹ |
| Versiani | 2005 | Main | 😐 | ☺ | ☺ | ☹ | ☺ | ☺ | 😐 | ☺ | ☺ | ☺ |
| Wheatley | 1998 | Main | 😐 | ☺ | ☺ | ☹ | ☺ | ☺ | ☺ | ☺ | ☺ | ☺ |
| WXL101497 | 2005 | Main | 😐 | ☺ | ☺ | ☹ | ☺ | 😐 | 😐 | ☺ | ☺ | ☺ |
| Zhao | 2006 | Main | 😐 | ☹ | ☹ | ☹ | ☹ | 😐 | 😐 | ☺ | ☺ | ☹ |
| De Nayer | 2002 | Spectrum of depressive disorders | 😐 | ☺ | ☺ | ☹ | ☺ | ☺ | 😐 | ☺ | ☺ | ☹ |
| Fava | 1998 | Spectrum of depressive disorders | 😐 | ☺ | ☺ | ☹ | ☺ | 😐 | 😐 | ☺ | ☺ | ☹ |
| Marchesi | 1998 | Spectrum of depressive disorders | 😐 | ☺ | ☺ | ☹ | ☺ | ☺ | ☹ | ☺ | ☺ | ☺ |
| Silverstone | 1999 | Spectrum of depressive disorders | 😐 | ☺ | ☺ | ☹ | ☺ | ☺ | 😐 | ☺ | ☺ | ☹ |
| Versiani | 1998 | Spectrum of depressive disorders | 😐 | ☺ | ☺ | ☹ | ☺ | 😐 | ☺ | ☺ | ☺ | ☹ |
| Ballus | 2000 | Spectrum of depressive disorders | 😐 | ☺ | ☺ | ☹ | ☺ | ☺ | 😐 | ☺ | ☺ | ☹ |
| Brasseur | 1989 | Spectrum of depressive disorders | 😐 | ☹ | ☹ | ☹ | ☹ | 😐 | 😐 | ☺ | ☺ | ☹ |
| Duarte | 1995 | Spectrum of depressive disorders | 😐 | ☺ | ☺ | ☹ | ☺ | ☺ | 😐 | ☺ | ☺ | ☹ |
| Lonnqvist | 1994 | Spectrum of depressive disorders | 😐 | ☺ | ☺ | ☹ | ☺ | ☺ | 😐 | ☺ | ☺ | ☹ |
| Serrano_Blanco | 2006 | Spectrum of depressive disorders | ☺ | ☺ | ☺ | ☺ | ☺ | ☺ | 😐 | ☺ | ☺ | ☺ |
| Simon | 1996 | Spectrum of depressive disorders | ☺ | ☹ | ☹ | ☹ | ☺ | 😐 | 😐 | ☺ | ☺ | ☹ |
| Vanelle | 1996 | Spectrum of depressive disorders | 😐 | ☺ | ☺ | ☹ | ☺ | 😐 | ☺ | ☺ | ☺ | ☺ |
| Judd | 2004 | Spectrum of depressive disorders | 😐 | ☺ | ☺ | ☹ | ☺ | ☺ | 😐 | ☺ | ☺ | ☹ |

**Observational studies**

| Author | Year | Analysis | Is the sample representative of patients in the population as a whole? | Are the patients at a similar point in the course of their condition/illness? | Are confounding factors identified and strategies to deal with them stated? | Was follow up carried out over a sufficient time period (6 months) ? | Are outcomes assessed using objective criteria? | Were the outcomes of people who withdrew described and included in the analysis? | Were outcomes measured in a reliable way? | Was appropriate statistical analysis used? |
| --- | --- | --- | --- | --- | --- | --- | --- | --- | --- | --- |
| De Jonghe | 1999 | Main | 😐 | 😐 | ☹ | ☹ | ☺ | ☹ | ☺ | ☹ |
| Fava | 1994 | Main | 😐 | 😐 | ☹ | ☹ | ☺ | ☹ | ☺ | ☹ |
| Fava | 1996 | Main | 😐 | 😐 | ☹ | ☹ | ☺ | ☹ | ☺ | ☹ |
| Nieremberg | 2000 | Main | 😐 | 😐 | 😐 | ☹ | ☺ | ☹ | ☺ | 😐 |
| Nieremberg | 1995 | Main | 😐 | 😐 | 😐 | ☹ | ☺ | ☹ | ☺ | ☺ |
| Baca_Baldomero | 2003 | Main | ☺ | ☹ | ☺ | ☺ | ☺ | ☹ | ☺ | ☹ |
| Cervera_Enguix | 2003 | Main | ☺ | ☹ | ☺ | ☺ | ☺ | ☹ | ☺ | ☹ |
| Dierick | 2002 | Main | 😐 | ☹ | ☺ | ☹ | ☺ | ☹ | ☺ | ☺ |
| Gonzales_Ruelas | 1997 | Main | 😐 | ☹ | ☹ | ☹ | ☺ | ☹ | ☺ | ☹ |
| Illescas_Rico | 2005 | Main | 😐 | ☹ | ☹ | ☹ | ☺ | ☹ | ☺ | ☹ |
| Vanderkooy | 2002 | Main | ☺ | ☹ | ☹ | ☹ | ☺ | ☹ | ☺ | ☹ |
| Wu | 2007 | Main | 😐 | 😐 | ☹ | ☺ | ☺ | ☹ | ☺ | ☹ |
| Dunner | 1997 | Spectrum of depressive disorders | ☺ | ☹ | 😐 | ☹ | ☺ | ☹ | ☺ | ☹ |
| Ravindran | 1998 | Spectrum of depressive disorders | 😐 | 😐 | ☹ | ☹ | ☺ | ☹ | ☺ | ☹ |
| Ros_Montalban | 2005 | Spectrum of depressive disorders | ☺ | ☹ | ☹ | ☹ | ☺ | ☺ | ☺ | ☺ |
| Sonawalla | 2002 | Spectrum of depressive disorders | 😐 | 😐 | ☹ | ☹ | ☺ | 😐 | ☺ | ☹ |
| Spatella | 2001 | Spectrum of depressive disorders | 😐 | ☹ | ☹ | ☹ | ☺ | 😐 | ☺ | ☹ |
| Perugi | 2002 | Spectrum of depressive disorders | 😐 | ☹ | ☹ | ☺ | ☺ | ☹ | ☺ | ☹ |
| Roca_Benassar | 2006 | Spectrum of depressive disorders | ☺ | ☹ | ☹ | ☺ | ☺ | ☹ | ☺ | ☹ |

**These two quality assessments were adapted from the standardized critical appraisal instruments from the Joanna Briggs Institute. For observational studies, we did not use one question which was not adapted because the question of the selection of cases was already dealt with and because there were no controls available in most of the studies: “***Has bias been minimised in relation to selection of cases and of controls?”***.**

☺ **Yes**

😐 **Unclear**

☹ **No**

**References**

1. Aguglia E, Casacchia M, Cassano GB, Faravelli C, Ferrari G, et al. (1993) Double-blind study of the efficacy and safety of sertraline versus fluoxetine in major depression. International clinical psychopharmacology 8: 197-202.

2. Akhondzadeh Basti A, Moshiri E, Noorbala AA, Jamshidi AH, Abbasi SH, et al. (2007) Comparison of petal of Crocus sativus L. and fluoxetine in the treatment of depressed outpatients: a pilot double-blind randomized trial. Prog Neuropsychopharmacol Biol Psychiatry 31: 439-442.

3. Akhondzadeh S, Faraji H, Sadeghi M, Afkham K, Fakhrzadeh H, et al. (2003) Double-blind comparison of fluoxetine and nortriptyline in the treatment of moderate to severe major depression. J Clin Pharm Ther 28: 379-384.

4. Akkaya C, Sivrioglu EY, Akgoz S, Eker SS, Kirli S (2006) Comparison of efficacy and tolerability of reboxetine and venlafaxine XR in major depression and major depression with anxiety features: an open label study. Hum Psychopharmacol 21: 337-345.

5. Alves C, Cachola I, Brandao J (1999) Efficacy and tolerability of venlafaxine and fluoxetine in outpatients with major depression. Primary Care Psychiatry 5: 57-63.

6. Amini H, Aghayan S, Jalili SA, Akhondzadeh S, Yahyazadeh O, et al. (2005) Comparison of mirtazapine and fluoxetine in the treatment of major depressive disorder: a double-blind, randomized trial. J Clin Pharm Ther 30: 133-138.

7. Amsterdam JD, Hooper MB, Amchin J (1998) Once- versus twice-daily venlafaxine therapy in major depression: a randomized, double-blind study. J Clin Psychiatry 59: 236-240.

8. Andreoli V, Caillard V, Deo RS, Rybakowski JK, Versiani M (2002) Reboxetine, a new noradrenaline selective antidepressant, is at least as effective as fluoxetine in the treatment of depression. J Clin Psychopharmacol 22: 393-399.

9. Ansseau M, Papart P, Troisfontaines B, Bartholomé F, Bataille M, et al. (1994) Controlled comparison of milnacipran and fluoxetine in major depression. Psychopharmacology 114: 131-137.

10. Baca Baldomero E, Cervera Enguix S (2003) [Quality of life, in depressed patients in Primary Health Care setting. Effectiveness and safety of venlafaxine extended release]. Actas Esp Psiquiatr 31: 331-338.

11. Badyal DK, Khosla PP, Deswal RS, Matreja PS (2006) Safety and efficacy of duloxetine versus venlafaxine in major depression in Indian patients. JK Science 8: 195-199.

12. Ballús C, Quiros G, De Flores T, de la Torre J, Palao D, et al. (2000) The efficacy and tolerability of venlafaxine and paroxetine in outpatients with depressive disorder or dysthymia. International clinical psychopharmacology 15: 43-48.

13. Beasley CM, Holman SL, Potvin JH (1993) Fluoxetine compared with imipramine in the treatment of inpatient depression. A multicenter trial. Annals of clinical psychiatry : official journal of the American Academy of Clinical Psychiatrists 5: 199-207.

14. Behnke K, Jensen GS, Graubaum HJ, Gruenwald J (2002) Hypericum perforatum versus fluoxetine in the treatment of mild to moderate depression. Adv Ther 19: 43-52.

15. Benkert O, Szegedi A, Philipp M, Kohnen R, Heinrich C, et al. (2006) Mirtazapine orally disintegrating tablets versus venlafaxine extended release: a double-blind, randomized multicenter trial comparing the onset of antidepressant response in patients with major depressive disorder. J Clin Psychopharmacol 26: 75-78.

16. Bennie EH, Mullin JM, Martindale JJ (1995) A double-blind multicenter trial comparing sertraline and fluoxetine in outpatients with major depression. J Clin Psychiatry 56: 229-237.

17. Berlanga C, Arechavaleta B, Heinze G, Campillo C, Torres M, et al. (1997) A double-blind comparison of nefazodone and fluoxetine in the treatment of depressed outpatients. Salud Mental 20: 1-8.

18. Besançon G, Cousin R, Guitton B, Lavergne F (1993) [Double-blind study of mianserin and fluoxetine in ambulatory therapy of depressed patients]. L'Encéphale 19: 341-345.

19. Bjerkenstedt L, Edman GV, Alken RG, Mannel M (2005) Hypericum extract LI 160 and fluoxetine in mild to moderate depression: a randomized, placebo-controlled multi-center study in outpatients. Eur Arch Psychiatry Clin Neurosci 255: 40-47.

20. Bowden CL, Schatzberg AF, Rosenbaum A, Contreras SA, Samson JA, et al. (1993) Fluoxetine and desipramine in major depressive disorder. Journal of clinical psychopharmacology 13: 305-311.

21. Boyer P, Danion JM, Bisserbe JC, Hotton JM, Troy S (1998) Clinical and economic comparison of sertraline and fluoxetine in the treatment of depression. A 6-month double-blind study in a primary-care setting in France. Pharmacoeconomics 13: 157-169.

22. Brasseur R (1989) A multicentre open trial of fluoxetine in depressed out-patients in Belgium. International clinical psychopharmacology 4 Suppl 1: 107-111.

23. Cervera-Enguix S, Soutullo CA, Landecho I, Murillo-Jelsbak R (2003) Quality of Life in 833 outpatients with major depression treated with open-label venlafaxine extended release: An observational 24-week study. pp. 193-197.

24. Chouinard G, Saxena B, Bélanger MC, Ravindran A, Bakish D, et al. (1999) A Canadian multicenter, double-blind study of paroxetine and fluoxetine in major depressive disorder. Journal of affective disorders 54: 39-48.

25. Clerc GE, Ruimy P, Verdeau-Pailles J (1994) A double-blind comparison of venlafaxine and fluoxetine in patients hospitalized for major depression and melancholia. International Clinical Psychopharmacology 9: 139-143.

26. Corne SJ, Hall JR (1989) A double-blind comparative study of fluoxetine and dothiepin in the treatment of depression in general practice. International clinical psychopharmacology 4: 245-254.

27. Corrigan MH, Denahan AQ, Wright CE, Ragual RJ, Evans DL (2000) Comparison of pramipexole, fluoxetine, and placebo in patients with major depression. Depress Anxiety 11: 58-65.

28. Costa e Silva J (1998) Randomized, double-blind comparison of venlafaxine and fluoxetine in outpatients with major depression. The Journal of clinical psychiatry 59: 352-357.

29. Cunningham LA (1997) Once-daily venlafaxine extended release (XR) and venlafaxine immediate release (IR) in outpatients with major depression. Venlafaxine XR 208 Study Group. Ann Clin Psychiatry 9: 157-164.

30. Cunningham LA, Borison RL, Carman JS, Chouinard G, Crowder JE, et al. (1994) A comparison of venlafaxine, trazodone, and placebo in major depression. J Clin Psychopharmacol 14: 99-106.

31. Dalery J, Honig A (2003) Fluvoxamine versus fluoxetine in major depressive episode: a double-blind randomised comparison. Hum Psychopharmacol 18: 379-384.

32. Dalery J, Rochat C, Peyron E, Bernard G (1992) [Comparative study of the efficacy and acceptability of amineptine and fluoxetine in patients with major depression]. L'Encéphale 18: 257-262.

33. De Jonghe F, Dekker J (1999) Early symptomatic changes in patients with major depression treated with antidepressants. The European journal of psychiatry 13: 69-76.

34. de Jonghe F, Ravelli DP, Tuynman Qua H (1991) A randomized, double-blind study of fluoxetine and maprotiline in the treatment of major depression. Pharmacopsychiatry 24: 62-67.

35. De Nayer A, Geerts S, Ruelens L, Schittecatte M, De Bleeker E, et al. (2002) Venlafaxine compared with fluoxetine in outpatients with depression and concomitant anxiety. Int J Neuropsychopharmacol 5: 115-120.

36. De Wilde J, Spiers R, Mertens C, Bartholomé F, Schotte G, et al. (1993) A double-blind, comparative, multicentre study comparing paroxetine with fluoxetine in depressed patients. Acta psychiatrica Scandinavica 87: 141-145.

37. Diaz-Martinez A, Benassinni O, Ontiveros A, Gonzalez S, Salin R, et al. (1998) A randomized, open-label comparison of venlafaxine and fluoxetine in depressed outpatients. Clin Ther 20: 467-476.

38. Dichter GS, Tomarken AJ, Freid CM, Addington S, Shelton RC (2005) Do venlafaxine XR and paroxetine equally influence negative and positive affect? J Affect Disord 85: 333-339.

39. Dierick M, De Nayer A, Ansseau M, D'Haenen H, Cosyns P, et al. (2002) An eight-week, open-label, uncontrolled, multicenter, phase IV study of remission rates in outpatients and inpatients with major depression treated with venlafaxine. pp. 475-485.

40. Dierick M, Ravizza L, Realini R, Martin A (1996) A double-blind comparison of venlafaxine and fluoxetine for treatment of major depression in outpatients. Prog Neuropsychopharmacol Biol Psychiatry 20: 57-71.

41. Duarte A, Mikkelsen H, Delini-Stula A (1996) Moclobemide versus fluoxetine for double depression: a randomized double-blind study. J Psychiatr Res 30: 453-458.

42. Dunner DL, Hendrickson HE, Bea C, Budech CB (1997) Venlafaxine in dysthymic disorder. J Clin Psychiatry 58: 528-531.

43. Fava M, Alpert J, Nierenberg AA, Mischoulon D, Otto MW, et al. (2005) A Double-blind, randomized trial of St John's wort, fluoxetine, and placebo in major depressive disorder. J Clin Psychopharmacol 25: 441-447.

44. Fava M, Amsterdam JD, Deltito JA, Salzman C, Schwaller M, et al. (1998) A double-blind study of paroxetine, fluoxetine, and placebo in outpatients with major depression. Ann Clin Psychiatry 10: 145-150.

45. Fava M, Bless E, Otto MW, Pava JA, Rosenbaum JF (1994) Dysfunctional attitudes in major depression. Changes with pharmacotherapy. J Nerv Ment Dis 182: 45-49.

46. Fava M, Davidson K, Alpert JE, Nierenberg AA, Worthington J, et al. (1996) Hostility changes following antidepressant treatment: Relationship to stress and negative thinking. pp. 459-467.

47. Fawcett J, Zajecka JM, Kravitz HM, Edwards J, Jeffriess H, et al. (1989) Fluoxetine versus amitriptyline in adult outpatients with major depression. Curr Ther Res, Clin Exp 45: 821-832.

48. Feighner JP, Boyer WF, Merideth CH, Hendrickson GG (1989) A double-blind comparison of fluoxetine, imipramine and placebo in outpatients with major depression. International clinical psychopharmacology 4: 127-134.

49. Feighner JP, Gardner EA, Johnston JA, Batey SR, Khayrallah MA, et al. (1991) Double-blind comparison of bupropion and fluoxetine in depressed outpatients. The Journal of clinical psychiatry 52: 329-335.

50. Ferreri M (1989) Fluoxetine versus amineptine in the treatment of outpatients with major depressive disorders. International clinical psychopharmacology 4 Suppl 1: 97-101.

51. Gagiano CA (1993) A double blind comparison of paroxetine and fluoxetine in patients with major depression. Br J Clin Res 4: 145-152.

52. Gattaz WF, Vogel P, Kick H, Kohnen R (1995) Moclobemide versus fluoxetine in the treatment of inpatients with major depression. J Clin Psychopharmacol 15: 35S-40S.

53. Geerts S, Bruynooghe F, De Cuyper H, Demeulemeester F, Haazen L (1994) Moclobemide versus fluoxetine for major depressive episodes. Clin Neuropharmacol 17 Suppl 1: S50-57.

54. Gentil V, Kerr-Correa F, Moreno R, D'Arrigo Busnello E, De Campos JA, et al. (2000) Double-blind comparison of venlafaxine and amitriptyline in outpatients with major depression with or without melancholia. J Psychopharmacol 14: 61-66.

55. Ginestet D (1989) Fluoxetine in endogenous depression and melancholia versus clomipramine. International clinical psychopharmacology 4 Suppl 1: 37-40.

56. Goldstein DJ, Mallinckrodt C, Lu Y, Demitrack MA (2002) Duloxetine in the treatment of major depressive disorder: a double-blind clinical trial. J Clin Psychiatry 63: 225-231.

57. Gonzalez Ruelas E, Diaz-Martinez A, Martinez Ruiz R (1997) An open assessment of the acceptability, efficacy, and tolerance of venlafaxine in usual care settings. pp. 609-630.

58. Gorenstein C, Andrade L, Moreno RA, Artes R (2002) Social adjustment in depressed patients treated with venlafaxine and amitriptyline. Int Clin Psychopharmacol 17: 171-175.

59. Guelfi JD, Ansseau M, Corruble E, Samuelian JC, Tonelli I, et al. (1998) A double-blind comparison of the efficacy and safety of milnacipran and fluoxetine in depressed inpatients. International clinical psychopharmacology 13: 121-128.

60. Guelfi JD, Van Hensbeek I, Ansseau M, Korsgaard S, Timmerman L (2000) Efficacy and tolerability of mirtazapine versus venlafaxine in hospitalized severely depressed patients with melancholia. International Journal of Neuropsychopharmacology 3: 245.

61. Guelfi JD, White C, Hackett D, Guichoux JY, Magni G (1995) Effectiveness of venlafaxine in patients hospitalized for major depression and melancholia. J Clin Psychiatry 56: 450-458.

62. Heiligenstein JH, Tollefson GD, Faries DE (1993) A double-blind trial of fluoxetine, 20 mg, and placebo in out-patients with DSM-III-R major depression and melancholia. International clinical psychopharmacology 8: 247-251.

63. Hong CJ, Hu WH, Chen CC, Hsiao CC, Tsai SJ, et al. (2003) A double-blind, randomized, group-comparative study of the tolerability and efficacy of 6 weeks' treatment with mirtazapine or fluoxetine in depressed Chinese patients. J Clin Psychiatry 64: 921-926.

64. Illescas-Rico R, Paez-Agraz F, Arreola-Chavez F, Verduzco-Fragoso W, Garcia-Rosas FA, et al. (2005) [Effectiveness and security of venlafaxine XR in the treatment of major depression]. Rev Med Inst Mex Seguro Soc 43: 473-478.

65. Joyce PR, Mulder RT, Luty SE, Sullivan PF, McKenzie JM, et al. (2002) Patterns and predictors of remission, response and recovery in major depression treated with fluoxetine or nortriptyline. Aust N Z J Psychiatry 36: 384-391.

66. Judd FK, Moore K, Norman TR, Burrows GD, Gupta RK, et al. (1993) A multicentre double blind trial of fluoxetine versus amitriptyline in the treatment of depressive illness. The Australian and New Zealand journal of psychiatry 27: 49-55.

67. Judd LL, Rapaport MH, Yonkers KA, Rush AJ, Frank E, et al. (2004) Randomized, placebo-controlled trial of fluoxetine for acute treatment of minor depressive disorder. Am J Psychiatry 161: 1864-1871.

68. Kennedy SH, Rizvi S, Fulton K, Rasmussen J (2008) A double-blind comparison of sexual functioning, antidepressant efficacy, and tolerability between agomelatine and venlafaxine XR. J Clin Psychopharmacol 28: 329-333.

69. Khan A, Upton GV, Rudolph RL, Entsuah R, Leventer SM (1998) The use of venlafaxine in the treatment of major depression and major depression associated with anxiety: a dose-response study. Venlafaxine Investigator Study Group. J Clin Psychopharmacol 18: 19-25.

70. Kuha S, Mehtonen OP, Henttonen A, Naarala M (1991) The efficacy of fluoxetine versus maprotiline in depressed patients and by dose. Nordisk Psykiatrisk Tidsskrift 45: 109-117.

71. Lapierre YD, Joffe R, McKenna K, Bland R, Kennedy S, et al. (1997) Moclobemide versus fluoxetine in the treatment of major depressive disorder in adults. J Psychiatry Neurosci 22: 118-126.

72. Lecrubier Y, Bourin M, Moon CA, Schifano F, Blanchard C, et al. (1997) Efficacy of venlafaxine in depressive illness in general practice. Acta Psychiatr Scand 95: 485-493.

73. Lee MS, Ham BJ, Kee BS, Kim JB, Yeon BK, et al. (2005) Comparison of efficacy and safety of milnacipran and fluoxetine in Korean patients with major depression. Curr Med Res Opin 21: 1369-1375.

74. Lemoine P, Guilleminault C, Alvarez E (2007) Improvement in subjective sleep in major depressive disorder with a novel antidepressant, agomelatine: randomized, double-blind comparison with venlafaxine. J Clin Psychiatry 68: 1723-1732.

75. Levine S, Deo R, Mahadevan K (1989) A comparative trial of a new antidepressant, fluoxetine. International clinical psychopharmacology 4 Suppl 1: 41-45.

76. Loeb C, Albano C, Gandolfo C (1989) Fluoxetine versus imipramine. International clinical psychopharmacology 4 Suppl 1: 75-79.

77. Lonnqvist J, Sintonen H, Syvälahti E, Appelberg B, Koskinen T, et al. (1994) Antidepressant efficacy and quality of life in depression: a double-blind study with moclobemide and fluoxetine. Acta psychiatrica Scandinavica 89: 363-369.

78. Lôo H, Saiz-Ruiz J, Costa e Silva J, Ansseau M, Herrington R, et al. (1999) Efficacy and safety of tianeptine in the treatment of depressive disorders in comparison with fluoxetine. Journal of affective disorders 56: 109-118.

79. Mao PX, Tang YL, Jiang F, Shu L, Gu X, et al. (2008) Escitalopram in major depressive disorder: a multicenter, randomized, double-blind, fixed-dose, parallel trial in a Chinese population. Depress Anxiety 25: 46-54.

80. Marchesi C, Ceccherininelli A, Rossi A, Maggini C (1998) Is anxious-agitated major depression responsive to fluoxetine? A double-blind comparison with amitriptyline. Pharmacopsychiatry 31: 216-221.

81. Martenyi F, Dossenbach M, Mraz K, Metcalfe S (2000) Gender differences in the antidepressive effect: A double-blind trial of fluoxetine and maprotiline in the treatment of major depression. European Neuropsychopharmacology 10: 221.

82. Massana J, Möller HJ, Burrows GD, Montenegro RM (1999) Reboxetine: a double-blind comparison with fluoxetine in major depressive disorder. International clinical psychopharmacology 14: 73-80.

83. McPartlin GM, Reynolds A, Andersen C, Casoy J (1998) A comparison of once-daily venlafaxine XR and paroxetine in depressed outpatients treated in general practice. Primary Care Psychiatry 4: 127-132.

84. Mehtonen OP, Søgaard J, Roponen P, Behnke K (2000) Randomized, double-blind comparison of venlafaxine and sertraline in outpatients with major depressive disorder. Venlafaxine 631 Study Group. The Journal of clinical psychiatry 61: 95-100.

85. Mendels J, Johnston R, Mattes J, Riesenberg R (1993) Efficacy and safety of b.i.d. doses of venlafaxine in a dose-response study. Psychopharmacology bulletin 29: 169-174.

86. Montgomery SA, Huusom AK, Bothmer J (2004) A randomised study comparing escitalopram with venlafaxine XR in primary care patients with major depressive disorder. Neuropsychobiology 50: 57-64.

87. Moreno RA, Teng CT, Almeida KM, Tavares Junior H (2005) Hypericum perforatum versus fluoxetine in the treatment of mild to moderate depression: a randomized double-blind trial in a Brazilian sample. Revista brasileira de psiquiatria 28: 29-32.

88. Nemeroff CB, Thase ME, Group ES (2007) A double-blind, placebo-controlled comparison of venlafaxine and fluoxetine treatment in depressed outpatients. Journal of psychiatric research 41: 351-359.

89. Nierenberg AA, Farabaugh AH, Alpert JE, Gordon J, Worthington JJ, et al. (2000) Timing of onset of antidepressant response with fluoxetine treatment. Am J Psychiatry 157: 1423-1428.

90. Nierenberg AA, McLean NE, Alpert JE, Worthington JJ, Rosenbaum JF, et al. (1995) Early nonresponse to fluoxetine as a predictor of poor 8-week outcome. Am J Psychiatry 152: 1500-1503.

91. Noguera R, Altuna R, Alvarez E, Ayuso JL, Casais L, et al. (1991) Fluoxetine vs. clomipramine in depressed patients: a controlled multicentre trial. J Affect Disord 22: 119-124.

92. Noorbala AA, Akhondzadeh S, Tahmacebi-Pour N, Jamshidi AH (2005) Hydro-alcoholic extract of Crocus sativus L. versus fluoxetine in the treatment of mild to moderate depression: a double-blind, randomized pilot trial. J Ethnopharmacol 97: 281-284.

93. Novotny V, Faltus F (2002) Tianeptine and fluoxetine in major depression: a 6-week randomised double-blind study. Hum Psychopharmacol 17: 299-303.

94. Ontiveros A G-BC (1997) A double-blind, comparative study of paroxetine and fluoxetine in out-patients with depression. British Journal of Clinical Research 823-32.

95. Ontiveros JB, F. Brunner, E. (1998) Estudio doble ciego sobre fluoxetina vs amitriptalina en los sintomas depressivos y de ansiedad Salud Mental 21: 58-63.

96. Owens MJ, Krulewicz S, Simon JS, Sheehan DV, Thase ME, et al. (2008) Estimates of serotonin and norepinephrine transporter inhibition in depressed patients treated with paroxetine or venlafaxine. Neuropsychopharmacology 33: 3201-3212.

97. Patris M, Bouchard JM, Bougerol T, Charbonnier JF, Chevalier JF, et al. (1996) Citalopram versus fluoxetine: a double-blind, controlled, multicentre, phase III trial in patients with unipolar major depression treated in general practice. International clinical psychopharmacology 11: 129-136.

98. Perugi G, Frare F, Toni C, Ruffolo G, Torti C (2002) Open-label evaluation of venlafaxine sustained release in outpatients with generalized anxiety disorder with comorbid major depression or dysthymia: effectiveness, tolerability and predictors of response. Neuropsychobiology 46: 145-149.

99. Rapaport M, Coccaro E, Sheline Y, Perse T, Holland P, et al. (1996) A comparison of fluvoxamine and fluoxetine in the treatment of major depression. J Clin Psychopharmacol 16: 373-378.

100. Ravindran AV, Charbonneau Y, Zaharia MD, al-Zaid K, Wiens A, et al. (1998) Efficacy and tolerability of venlafaxine in the treatment of primary dysthymia. J Psychiatry Neurosci 23: 288-292.

101. Remick RA, Claman J, Reesal R, Gibson RE, Agbayewa MO, et al. (1993) Comparison of fluoxetine and desipramine in depressed outpatients. Curr Ther Res Clin Exp 53: 457-465.

102. Remick RA, Keller FD, Gibson RE, Carter D (1989) A comparison between fluoxetine and doxepin in depressed patients. Curr Ther Res, Clin Exp 46: 842-848.

103. Reynaert C, Parent M, Mirel J, Janne P, Haazen L (1995) Moclobemide versus fluoxetine for a major depressive episode. Psychopharmacology (Berl) 118: 183-187.

104. Roca Benassar M, Baca Baldomero E (2006) [Response and remission in depressive patients with anxiety symptoms treated with venlafaxine extended release in primary care]. Actas Esp Psiquiatr 34: 162-168.

105. Ropert R (1989) Fluoxetine versus clomipramine in major depressive disorders. International clinical psychopharmacology 4 Suppl 1: 89-95.

106. Ros Montalban S, Mora Ripoll R, Garcia-Garcia M (2005) [The current challenges of the treatment of depression: venlafaxina extended release and remission outcomes in real-world clinical practice]. Actas Esp Psiquiatr 33: 165-172.

107. Rudolph RL, Fabre LF, Feighner JP, Rickels K, Entsuah R, et al. (1998) A randomized, placebo-controlled, dose-response trial of venlafaxine hydrochloride in the treatment of major depression. J Clin Psychiatry 59: 116-122.

108. Rudolph RL, Feiger AD (1999) A double-blind, randomized, placebo-controlled trial of once-daily venlafaxine extended release (XR) and fluoxetine for the treatment of depression. J Affect Disord 56: 171-181.

109. Samuelian JC, Hackett D (1998) A randomized, double-blind, parallel-group comparison of venlafaxine and clomipramine in outpatients with major depression. J Psychopharmacol 12: 273-278.

110. Sauer H, Huppertz-Helmhold S, Dierkes W (2003) Efficacy and safety of venlafaxine ER vs. amitriptyline ER in patients with major depression of moderate severity. Pharmacopsychiatry 36: 169-175.

111. Schrader E (2000) Equivalence of St John's wort extract (Ze 117) and fluoxetine: a randomized, controlled study in mild-moderate depression. Int Clin Psychopharmacol 15: 61-68.

112. Schweizer E, Feighner J, Mandos LA, Rickels K (1994) Comparison of venlafaxine and imipramine in the acute treatment of major depression in outpatients. J Clin Psychiatry 55: 104-108.

113. Sechter D, Troy S, Paternetti S, Boyer P (1999) A double-blind comparison of sertraline and fluoxetine in the treatment of major depressive episode in outpatients. Eur Psychiatry 14: 41-48.

114. Serrano-Blanco A, Gabarron E, Garcia-Bayo I, Soler-Vila M, Caramés E, et al. (2006) Effectiveness and cost-effectiveness of antidepressant treatment in primary health care: a six-month randomised study comparing fluoxetine to imipramine. Journal of affective disorders 91: 153-163.

115. Sheehan DV, Nemeroff CB, Thase ME, Entsuah R (2009) Placebo-controlled inpatient comparison of venlafaxine and fluoxetine for the treatment of major depression with melancholic features. pp. 61-86.

116. Shelton RC, Haman KL, Rapaport MH, Kiev A, Smith WT, et al. (2006) A randomized, double-blind, active-control study of sertraline versus venlafaxine XR in major depressive disorder. J Clin Psychiatry 67: 1674-1681.

117. Silverstone PH, Ravindran A (1999) Once-daily venlafaxine extended release (XR) compared with fluoxetine in outpatients with depression and anxiety. Journal of Clinical Psychiatry 60: 22-28.

118. Simon GE, VonKorff M, Heiligenstein JH, Revicki DA, Grothaus L, et al. (1996) Initial antidepressant choice in primary care. Effectiveness and cost of fluoxetine vs tricyclic antidepressants. JAMA : the journal of the American Medical Association 275: 1897-1902.

119. Sir A, D'Souza RF, Uguz S, George T, Vahip S, et al. (2005) Randomized trial of sertraline versus venlafaxine XR in major depression: efficacy and discontinuation symptoms. J Clin Psychiatry 66: 1312-1320.

120. Sonawalla SB, Farabaugh A, Johnson MW, Morray M, Delgado ML, et al. (2002) Fluoxetine treatment of depressed patients with comorbid anxiety disorders. J Psychopharmacol 16: 215-219.

121. Spalletta G, Pasini A, Caltagirone C (2002) Fluoxetine alone in the treatment of first episode anxious-depression: an open clinical trial. J Clin Psychopharmacol 22: 263-266.

122. Suleman MI, Sebit MB, Acuda SW, Siziya S (1997) Fluoxetine and moclobemide versus amitriptyline in major depression: A single blind randomized clinical trial in Zimbabwe. Central African Journal of Medicine 43: 38-40.

123. Suri RA, Altshuler LL, Rasgon NL, Calcagno JL, Frye MA, et al. (2000) Efficacy and response time to sertraline versus fluoxetiae in the treatment of unipolar major depressive disorder. Journal of Clinical Psychiatry 61: 942-946.

124. Tamminen TT, Lehtinen VV (1989) A double-blind parallel study to compare fluoxetine with doxepin in the treatment of major depressive disorders. International clinical psychopharmacology 4 Suppl 1: 51-56.

125. Thase ME (1997) Efficacy and tolerability of once-daily venlafaxine extended release (XR) in outpatients with major depression. The Venlafaxine XR 209 Study Group. J Clin Psychiatry 58: 393-398.

126. Thase ME, Clayton AH, Haight BR, Thompson AH, Modell JG, et al. (2006) A double-blind comparison between bupropion XL and venlafaxine XR: sexual functioning, antidepressant efficacy, and tolerability. J Clin Psychopharmacol 26: 482-488.

127. Tignol J (1993) A double-blind, randomized, fluoxetine-controlled, multicenter study of paroxetine in the treatment of depression. Journal of clinical psychopharmacology 13: 18s-22s.

128. Tollefson GD, Greist JH, Jefferson JW, Heiligenstein JH, Sayler ME, et al. (1994) Is baseline agitation a relative contraindication for a selective serotonin reuptake inhibitor: a comparative trial of fluoxetine versus imipramine. J Clin Psychopharmacol 14: 385-391.

129. Tural U, Onder E (2003) Fluoxetine once every third day in the treatment of major depressive disorder. Eur Arch Psychiatry Clin Neurosci 253: 307-312.

130. Tylee A, Beaumont G, Bowden MW, Reynolds A (1997) A double-blind, randomized, 12-week comparison study of the safety and efficacy of venlafaxine and fluoxetine in moderate to severe major depression in general practice. Primary Care Psychiatry 3: 51-58.

131. Tzanakaki M, Guazzelli M, Nimatoudis I, Zissis NP, Smeraldi E, et al. (2000) Increased remission rates with venlafaxine compared with fluoxetine in hospitalized patients with major depression and melancholia. Int Clin Psychopharmacol 15: 29-34.

132. Van Moffaert M, Bartholome F, Cosyns P, De Nayer AR, Mertens C (1995) A controlled comparison of sertraline and fluoxetine in acute and continuation treatment of major depression. Human Psychopharmacology: Clinical and Experimental 10: 393-405.

133. Vanderkooy JD, Kennedy SH, Bagby RM (2002) Antidepressant side effects in depression patients treated in a naturalistic setting: A study of bupropion, moclobemide, paroxetine, sertraline, and venlafaxine. Canadian Journal of Psychiatry Revue Canadienne de Psychiatrie 47: 174-180.

134. Vanelle JM, Attar-Levy D, Poirier MF, Bouhassira M, Blin P, et al. (1997) Controlled efficacy study of fluoxetine in dysthymia. Br J Psychiatry 170: 345-350.

135. Versiani M, Moreno R, Ramakers-van Moorsel CJ, Schutte AJ, Comparative Efficacy Antidepressants Study G (2005) Comparison of the effects of mirtazapine and fluoxetine in severely depressed patients. CNS drugs 19: 137-146.

136. Versiani M, Ontiveros A, Mazzotti G, Ospina J, Dávila J, et al. (1999) Fluoxetine versus amitriptyline in the treatment of major depression with associated anxiety (anxious depression): a double-blind comparison. International clinical psychopharmacology 14: 321-327.

137. Wheatley DP, van Moffaert M, Timmerman L, Kremer CM (1998) Mirtazapine: efficacy and tolerability in comparison with fluoxetine in patients with moderate to severe major depressive disorder. Journal of Clinical Psychiatry 59: 306-312.

138. Wu YS, Chen YC, Lu RB (2007) Venlafaxine vs. paroxetine in the acute phase of treatment for major depressive disorder among Han Chinese population in Taiwan. J Clin Pharm Ther 32: 353-363.

139. Zhao J, Ang Q, Wang J, Sun X, Wei J, et al. (2006) A comparative efficacy of fluoxetine and trazodone in depression with remarkable retardation and loss of energy: A randomized open-label trial. International Medical Journal 13: 25-30.
